# Calibration-free infrared absorption spectroscopy using cantilever-enhanced photoacoustic detection of the optical power

**DOI:** 10.1016/j.pacs.2024.100655

**Published:** 2024-10-09

**Authors:** Jussi Rossi, Markku Vainio

**Affiliations:** a Photonics Laboratory, Physics Unit, Tampere University, Tampere, Finland; b Department of Chemistry, University of Helsinki, Helsinki, Finland

**Keywords:** Tunable diode laser absorption spectroscopy, Cantilever-enhanced photoacoustic spectroscopy, Trace gas sensing

## Abstract

We report on sensitive tunable laser absorption spectroscopy using a multipass gas cell and a solid-state photoacoustic optical power detector. Unlike photoacoustic spectroscopy (PAS), this method readily allows a low gas pressure for high spectral selectivity and a free gas flow for continuous measurements. Our photoacoustic optical power detector has a large linear dynamic range and can be used at almost any optical wavelength, including the middle infrared and THz regions that are challenging to cover with traditional optical detectors. Furthermore, our approach allows for compensation of laser power drifts with a single detector. As a proof of concept, we have measured very weak CO_2_ absorption lines at 9.2 µm wavelength and achieved a normalized noise equivalent absorption (NNEA) of 2.35·10^−9^ Wcm^−1^Hz^−1/2^ with a low-power quantum cascade laser. The absolute value of the gas absorption coefficient is obtained directly from the Beer-Lambert law, making the technique calibration-free.

## 1 Introduction

Traditionally, the photoacoustic (PA) effect is used in laser spectroscopy of gases “directly”. That is, the photoacoustic signal is generated through absorption in the target gas that is placed inside of a PA cell, where a highly sensitive microphone detects the pressure change originating from the non-radiative relaxation of the target molecules. State-of-the-art examples include cantilever-enhanced photoacoustic spectroscopy (CEPAS) [1], [2] and quartz-enhanced photoacoustic spectroscopy (QEPAS) [3], [4], [5]. It is also possible to utilize the PA effect by using a solid-state PA detector as an optical power detector to measure the transmission spectrum, i.e., power attenuation experienced by a laser light beam after it has passed through a separate gas cell. In other words, the photodetector of conventional tunable diode laser absorption spectroscopy (TDLAS) is replaced by a PA power detector. Here, we demonstrate this with a cantilever-enhanced photoacoustic power detector, where the optical power incident on the detector is absorbed by a black absorber (e.g., soot) and the resulting acoustic signal is recorded by a highly sensitive cantilever microphone [6]. The cantilever-enhanced optical power detector has a large linear dynamic range, it can be operated at room temperature, and it works at any optical wavelength from ultraviolet (UV) to THz [7], [8]. As demonstrated in this paper, this enables selective and sensitive gas detection, for example, in the ∼ 7–20 µm fingerprint region, where many molecules have strong rovibrational transitions. As an example, benzene has its strongest rotational-vibrational transitions at 15 µm, which is inaccessible by state-of-the-art infrared photodetectors. Currently, sensitive measurements at such long wavelengths are typically done using photoacoustic spectroscopy [9].

While traditional PA-spectroscopy is known for its excellent detection sensitivity (if high laser power is available), it has its limitations. The main limitations are the need for calibration of the absorption measurement and the need for a relatively high gas pressure. These problems can often be avoided in TDLAS, where the detection is separated from the gas cell. With a separate gas cell, continuous measurements are possible even with flowing gases and the gas pressure can be chosen freely. Low pressure is often preferred because reduced collisional broadening improves spectroscopic resolution and selectivity. At low pressures, even Doppler-free saturation spectroscopy experiments become possible [10]. These are generally not possible in PAS because gas flow interferes with the microphone and the reduction of pressure affects the propagation of the pressure wave, reducing the signal strength. Furthermore, in PAS, the signal generation strongly depends on the target gas, transition, as well as the gas matrix. This is a result of different molecular relaxation pathways and can lead even to complete nullification of the PA signal [11].

On the other hand, with TDLAS, the zero-background property of PAS is lost, making it more difficult to achieve high detection sensitivity, as a small signal needs to be measured against a large background. This calls for a detector with low technical noise and high dynamic range, and access to a spectral region of strong molecular absorption. Wavelength and frequency modulation techniques [12], [13] can be used to improve the detection sensitivity, however, at the cost of lost absoluteness of absorption measurement. Another method for sensitivity enhancement is to increase absorption path length, for example using a multipass cell. In this case, if the transmission signal is measured directly, the absolute value of absorption can be deduced from the Beer-Lambert law.

In this paper, we present a method that combines a major advantage of photoacoustic detection (operation at any wavelength) with benefits of TDLAS (versatility, absoluteness). For our demonstration, we chose to use a multipass cell, which is commonly applied in trace-gas spectroscopy due to its well-known benefits [14], [15], [16], [17], [18]. Using a 76-m long astigmatic Herriot multipass cell and a cantilever-enhanced optical power detector, we have obtained a detection sensitivity comparable to direct PAS, however using a low laser power (∼ 20 mW). Importantly, owing to the direct transmission spectrum measurement, the absorption can be determined without calibration, and low sample gas pressure can be used for high spectral resolution.

A method similar to the one presented here is light-induced thermoelastic spectroscopy (LITES), which has been extensively investigated during the past few years [19], [20], [21], [22], [23], [24], [25], [26], [27]. Both methods are based on TDLAS, i.e., on monitoring of the optical power transmitted through a separate gas volume, often with a multipass cell. However, the principle of detector signal formation is very different. In our case, the optical power is measured with a photoacoustic detector based on a cantilever microphone, while in LITES, the signal is a piezoelectric current caused by thermoelastic expansion of a quartz tuning fork (QTF) upon illumination.

## 2 Spectrometer design

**Fig. 1:**
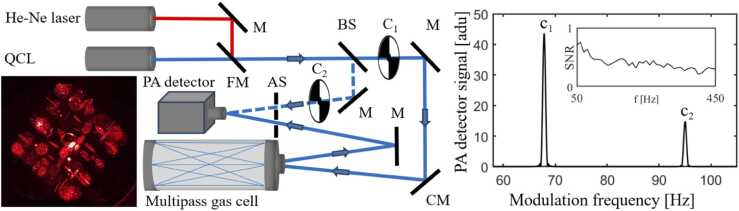
A simplified schematic of the TDLAS measurement setup. The laser beam is divided into two arms with a beamsplitter (BS). The transmitted part (signal beam) is focused with a concave mirror (CM) to the center of the multipass gas cell. After the cell, the beam is guided to a photoacoustic (PA) power detector. The reference beam (dashed line) is reflected to the PA detector with a mirror (M). The aperture stop (AS) is placed in front of the detector to avoid stray radiation. The signal and reference beams are modulated with optical choppers C_1_ and C_2_, respectively. An example of the respective PA detector signal is plotted in arbitrary detector units (adu) on the right and the signal-to-noise ratio (SNR) of the PA detector as a function of modulation frequency in the inset. For alignment purposes, a He-Ne laser beam was reflected with a flip mirror (FM) to the same optical path as the QCL. Lissajous pattern (photographed with He-Ne laser) on the surface of the astigmatic end mirror is presented on the left.

The spectrometer used in this work is shown schematically in Fig. 1. The light source is a wavelength-tunable quantum cascade laser, QCL (AdTech optics, DFB/ HHL-13–48, 9.2 µm), which is operated with a laser current and temperature controller (Thorlabs ITC4002QCL). The laser output beam is first modulated with a mechanical chopper, and then guided into an astigmatic Herriot multipass cell (Aerodyne AMAC-76). The same multipass cell design is commonly used in high-precision trace gas detection and can also be applied to measurements with fast gas flows without causing significant interference on the beam path [14], [15], [16], [17], [18]. The beam transmitted through the cell is forwarded to the PA power detector. The power detector signal is digitized, and Fourier transformed into a power spectrum with a software implemented in NI LabVIEW. An example of the recorded power signal is shown in Fig. 1, which also shows the typical chopping frequencies used in our experiments (of the order of tens of Hz). Note that the height of a signal peak is directly proportional to the optical power received at the detector.

To compensate for long-term drifts in laser power, we applied a dual-beam detection scheme that can be implemented with a single PA power detector: the laser beam is divided into two arms with a beam splitter, and each arm is chopped at a distinct frequency. One of the modulated beams (“signal beam”) passes through the gas cell, while the other is used as a reference. Both beams are finally guided to the same detector. The broad acoustic frequency span of the cantilever-enhanced detector enables simultaneous detection of both signals (c_1_ and c_2_ in Fig. 1), and the transmission spectrum is obtained as a ratio of them. The modulation frequencies, Fourier time constant (signal recording time) and signal apodization were optimized to minimize crosstalk between the two signals.

Detailed description of the photoacoustic detector and its properties, including the dynamic range, linearity and sensitivity with different absorbers can be found in our previous reports [6], [7]. Here, we used soot as the absorber material. The multipass cell consists of two astigmatic mirrors (one having a central coupling hole) that are placed 0.32 cm apart inside a sealed pyrex tube, which has a gas volume of 0.5 l. Light is directed in and out through the same BaF_2_ window with 3.2° in-out half-angle. Since the input hole diameter is only 4.3 mm, we focused the laser beam to the center of the cell to avoid beam clipping. With the laser beam properly aligned, the beam goes through 238 passes, which makes the total absorption path length 76 m.

## 3 Results

In order to characterize the spectrometer performance, we measured weak CO_2_ absorption lines in the 9.2 µm wavelength region. The intensities of these absorption lines are only of the order of 10^−24^ cm^−1^/(molecule·cm^−2^) [28] which allowed simple and reliable system characterisation without the challenge of generating highly diluted gas mixtures.

**Fig. 2:**
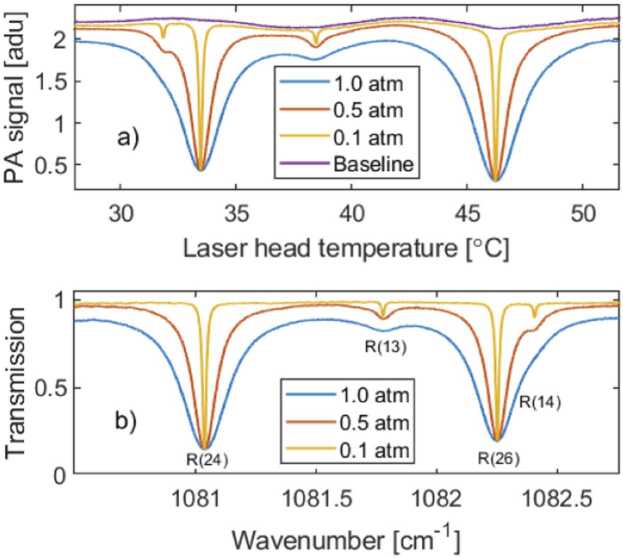
(a) The raw spectra in arbitrary detector units (adu) of a CO_2_/N_2_ gas mixture measured at different pressures by scanning the laser temperature, using the 76-m long multipass cell. The baseline was recorded with pure N_2_ at 1 atm. The transmission spectra shown in (b) are background-corrected (divided by the baseline). The Fourier time constant of detection was set to *T* = 783 ms and a time delay of 800 ms was used between 0.01 °C steps. Total measurement time of each scan was ∼29 min. The strongest lines R(24) and R(26) belong to the 00011–10002 band, while the weaker lines R(13) and R(14) are of the 01111–11102 band of ^12^C^16^O_2_[28].

**Fig. 3:**
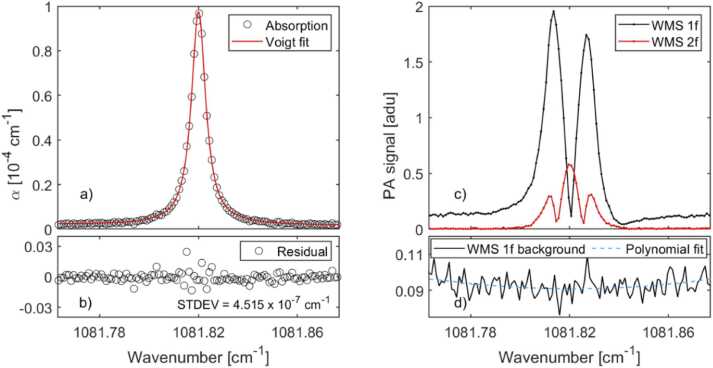
Measurements of line R(13) with pure CO_2_ at 0.042 atm pressure and with 76 m path length, obtained by scanning the laser current. (a) Absorption peak calculated from the direct TDLAS measurement and a Voigt profile fitted to it. (b) The residual of (a), i.e., the deviation of the measured points from the Voigt fit. (c) The same measurement with 1 f and 2 f WMS given in arbitrary detector units (adu). (d) Baseline of the 1 f signal and a polynomial fit to it. As for the Voigt fit of (a), full-width-at-half-maximum linewidths of 0.00095 cm^−1^ and 0.00683 cm^−1^ were obtained for the Gaussian and Lorentzian components, respectively. The predicted values are 0.001 cm^−1^ (calculated Doppler width) and 0.00865 cm^−1^ (self-broadening, HITRAN [28]).

The transmission spectra were recorded by scanning the wavelength via either laser temperature or current. Temperature scan was used for recording of a wider spectrum (Fig. 2). Current scan provides a higher precision and was used to record a narrow single peak (Fig. 3). To avoid complexity in data analyzing, only one parameter was scanned at a time. Chopping frequencies of 120 Hz and 147 Hz were used for the signal (c_1_ in Fig. 1) and reference (c_2_) beams, respectively. These frequencies were chosen to minimize crosstalk of the signals. The detector’s noise level is mainly 1/f dependent, but so is the response [6]. Note that, unlike the tuning-fork design of LITES [22], [26], our detector is not based on an acoustic resonance. As a result, the signal-to-noise ratio varies only slightly between 50 and 450 Hz (Fig. 1 figure inset). This means that the measurement frequencies can be chosen quite freely and several modulation frequencies can be used simultaneously.

Examples of laser temperature scans are shown in Fig. 2 for different gas pressures. Here, a mixture of CO_2_ (∼ 10 %) and N_2_ (∼ 90 %) was used to avoid saturation of the absorption. Because the BaF_2_ window of the multipass cell caused optical interference fringes in the spectrum (the period of fringes matches the free spectral range calculated for the window), we also measured the baseline by performing the same scan with pure N_2_ (the purple line in Fig. 2a). The final transmission spectra (Fig. 2b) were obtained by dividing the spectra of Fig. 2a with the baseline. The appearance of interference fringes is a typical problem with multipass cells [18], [29], but in our setup the fringes were found to be stable and predictable, and did not cause any uncertainties in long-term measurements. The wavenumber axes were linearized with a wavelength meter (Bristol 771B). The absorption line center wavenumbers agree with those found in the HITRAN database [28].

In order to evaluate the detection limit of the spectrometer, we then used pure CO_2_ at low pressure (0.042 atm) and measured the weak peak R(13) at 1081.82 cm^−1^ by scanning the laser current (Fig. 3a). Here, only the signal through the multipass cell (c_1_ at 120 Hz, Fig. 1) was recorded without a reference beam. This was done because, as explained below, the dual-beam detection turned out to be useful only for long measurement times and the laser current scans were relatively fast. Each scan was done by increasing the laser current in 32 µA steps from 532 mA to 545 mA with 200 ms time delay, leading to a total measurement time of ∼1 min. The Fourier time constant of detection was set to *T* = 392 ms.

The absorption coefficient *α* was calculated from the baseline-corrected transmission spectrum with the Beer-Lambert law *α = -ln(I/I*_*0*_*)/L*, where *I/I*_*0*_ is the transmission. Voigt profile was fitted to the absorption peak (Fig. 3a). The standard deviation of the fit residual (Fig. 3b) gives an estimate for the noise equivalent absorption (NEA), 4.52·10^−7^ cm^−1^. To calculate the NNEA, this was divided by 1.5·*T*^−1^, where *T* is the Fourier time constant and the factor of 1.5 accounts for the equivalent noise bandwidth for Hanning apodizing function [30]. Furthermore, we calculated the power-normalized NEA to enable comparison with PAS methods. The optical power used for normalization (21.2 mW) was measured from the input of the multipass cell at the center wavenumber of the absorption peak. (The total optical losses of the multipass were measured to be 86 %. Therefore, a maximum of 0.14·21.2 mW = 2.9 mW reached the PA power detector.)

A summary of the calculated N(NEA) values is given in Table 1.

**Table 1:** Quantitative comparison of different modulation techniques. Modulation frequency of 120 Hz and *T* = 392 ms were used for all but the MCT measurement (see text for details). The optical power incident to the multipass cell (and used for normalization) was 21.2 mW in all measurements.

	**SNR**	**NEA** **[10**^**−7**^**cm**^**−1**^**]**	**NNEA** **[10**^**−7**^**cm**^**−1**^**Hz**^**−1/2**^**]**	**NNEA** **[10**^**−9**^**Wcm**^**−1**^**Hz**^**−1/2**^**]**
**PA (chopped)**	215	4.52	2.31	4.90
**PA WMS 1 f**	303	2.17	1.11	2.35
**PA WMS 2 f**	290	2.27	1.16	2.46
**MCT**	101	8.82	3.44	7.28

For comparison, we also tested wavelength modulation spectroscopy (WMS) [3], [12], which was implemented by modulating the laser wavelength instead of chopping its power. The wavelength modulation was done by modulating the laser current with a sinusoidal signal at 120 Hz. The modulation amplitude was optimized to maximize the WMS signal strength [12], and both the first and second harmonic (1 f and 2 f) signals were simultaneously recorded from the Fourier-transformed PA power detector signal using Fourier time constant *T* = 392 ms.

The WMS measurements were calibrated using a direct absorption measurement performed with the same gas sample in the same conditions (pressure 0.042 atm, pure CO_2_). The transmission peak contrast of the direct measurement (0.50) was then used to calculate the NNEA values for WMS. The baseline of the 1 f WMS signal was corrected by dividing it with its polynomial fit function (Fig. 3d). For 2 f WMS, there was no need for correction owing to the inherently flat baseline.

To compare our approach with an implementation based on a state-of-the-art infrared photodetector, we also carried out direct transmission measurements by replacing the PA power detector with a Peltier-cooled Mercury-Cadmium-Telluride (MCT) detector (Vigo PEM-10.6). (However, note that the MCT detector cannot be used at wavelengths longer than ∼12 µm [31], for which reason we chose the 9 µm spectral region for the comparison.) In this case, no chopping or other modulation was needed. The detection bandwidth was reduced with a low-pass filter for consistent comparison with the PA power detector. The –3 dB bandwidth of the (first-order) filter was 4.2 Hz, which corresponds to an equivalent noise bandwidth of 6.6 Hz (cf. 3.8 Hz of PA detection with Hanning apodization and *T* = 392 ms) [30]. Although the equivalent noise bandwidth was taken into account in NNEA calculation, this makes a slightly unfair comparison because the MCT detectors are designed for higher measurement frequencies. However, since there are only a few high-quality detector types available for the longer infrared, the comparison gives valid information of different detector techniques. Wavelength-modulation spectroscopy with MCT would have been an interesting additional test for comparison, but the measurement was limited by the digitization noise of the lock-in amplifier needed for signal demodulation. With the PA power detector, this issue was avoided due to the higher dynamic range of data acquisition, which is based on direct optical readout of the cantilever (photoacoustic sensor), followed by fast Fourier transform [7].

**Fig. 4:**
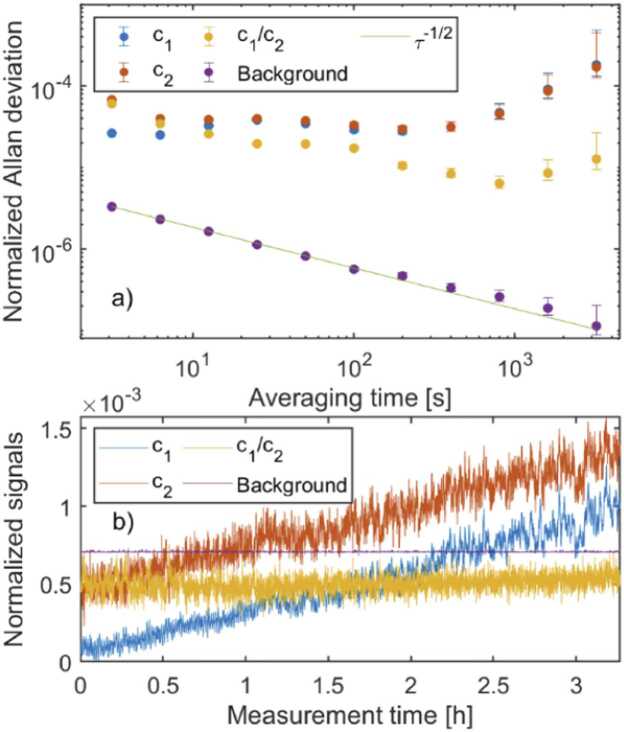
(a) Normalized Allan deviations of c_1_ (signal channel), c_2_ (reference channel) and c_1_/c_2._ (dual-beam detection) when the multipass cell is bypassed with a gold mirror. *T* = 3.13 s. Background was measured by blocking the laser beam and τ^−1/2^ indicates the theoretical white-noise slope of Allan deviation. (b) Normalized raw signals used in the calculations of Allan deviations shown in (a). The signal offsets are adjusted to fit all plots in the same figure.

The capacity of dual-beam detection to compensate for fluctuations and drifts in laser power was tested by bypassing the multipass cell with a mirror. Both chopped laser beams (corresponding to signals c_1_ and c_2_, see Fig. 1) were reflected directly to the PA power detector and data were recorded for 3 hours with *T* = 3130 ms Fourier time constant. The data were analyzed with AlaVar5 freeware program and Allan deviations were calculated for signals c_1_ and c_2_, as well as for their ratio c_1_/c_2_ (dual-beam detection). The background noise of the detector was recorded with the laser beam blocked. The normalizations in Figs. 4a and 4b were done by dividing the recorded raw signals with their time-averaged values.

Fig. 4a shows that the dual-beam detection method reduces the long-term drifts by almost a decade with 13 min averaging time (blue and yellow dots). However, the compensation does not work for short-term fluctuations at times scales of the order of *T*, which can be seen by comparing the blue and yellow plots in Fig. 4b. The PA power detector background noise averages nicely and follows the theoretical white-noise slope as assumed, leaving room for more than an order of magnitude improvement in gas detection sensitivity and long-term stability.

## 4 Conclusions

In this work, a gas detection method based on TDLAS with a 76-m multipass cell and a room-temperature solid-state photoacoustic optical power detector was reported. Compared to traditional photoacoustic methods, this approach has advantages: the absolute value of absorption is obtained without calibration, and low gas pressure can be used for high spectral selectivity. Furthermore, since the acoustic sensor is physically separated from the gas volume under study, free gas flow can be used for continuous measurements, for instance, with multipass cells [32], [33]. The use of photoacoustic optical power detection thus enables high-precision TDLAS measurements to be extended to the long-wavelength side of the molecular fingerprint region (7–20 µm), well beyond the upper limit of MCT detectors. Even though our method is introduced here for the first time, the demonstrated NNEA of 4.90·10^−9^ Wcm^−1^Hz^-½^ is comparable with other state-of-the-art photoacoustic and photothermal gas detection methods, which, however, generally require calibration. As an example, typical NNEA values for LITES are (1−100)·10^−9^ Wcm^−1^Hz^−1/2^ [22], [23], [24] and the lowest reported value found from the literature is 0.916·10^−9^ Wcm^−1^Hz^−1/2^ [25]. With QEPAS and CEPAS, which are also suitable for high-precision spectroscopy and sensing in the long-wavelength fingerprint region, values down to the 0.26·10^−9^ Wcm^−1^Hz^−1/2^ level have been reported [3], [5], [34]. (With additional cavity enhancement, further improvement down to ∼1.7·10^−12^ Wcm^−1^Hz^−1/2^ are achievable with both QEPAS and CEPAS [2], [35].) It is worth noting that excellent NNEA values can generally be obtained with cavity-enhanced spectroscopy methods, but those methods are limited to shorter wavelengths due to the lack of suitable detector and mirror technologies [10].

We achieved further improvement in NNEA (down to 2.35·10^−9^ Wcm^−1^Hz^−1/2^) by applying 1 f wavelength modulation spectroscopy, which is less prone to laser intensity noise. This corresponds to the lowest detectable volume mixing ratio of 0.0033 (1σ) with the weak CO_2_ line used here (spectral line intensity of 1.42·10^−24^ cm^−24^/(molecule·cm^−2^)). This means that a limit of detection of ∼1 ppb could be achieved with the same experimental parameters if the strong fundamental rovibrational lines were targeted instead; as an example, the CO_2_ lines at 4.28 µm have line intensities of the order of 3.3·10^−18^ cm^−1^/(molecule·cm^−2^) [28].

While detection sensitivity is a key factor in trace-gas spectroscopy, other features are also important. For example, the surface area of our power detector is large (with a diameter of 1 cm), making it readily applicable at long infrared and THz wavelengths, where laser beam sizes are inherently large. This is a significant advantage compared to, e.g., LITES, where the laser beam needs to be focused precisely to a small target point at the root of a tuning fork, which is typically a few millimeters in size [27]. Furthermore, our power detector offers excellent detection sensitivity without the use of acoustic resonance. This unique feature allows for the use of multiple modulation frequencies simultaneously, enabling measurements at multiple wavelengths or, as demonstrated here, compensation for laser power drifts using a single detector. In general, our method provides a combination of high detection sensitivity, precision, and spectral selectivity, making it applicable to measurements of atmospheric trace gases, pollutants or isotopic ratios [16]. Owing to the photoacoustic optical power detector design, the method is suitable for any wavelength between UV and THz. In particular, it enables enhanced detection sensitivity and selectivity for important trace-gas molecules in the molecular fingerprint region.

## Funding

The work was funded by the 10.13039/501100002341Academy of Finland (Project number 326444) and by the Academy of Finland Flagship Programme, Photonics Research and Innovation (PREIN), decision numbers: 320167 and 320165. J. Rossi acknowledges the financial support of The 10.13039/501100003125Finnish Cultural Foundation.

## CRediT authorship contribution statement

**Markku Vainio:** Writing – review & editing, Supervision, Resources, Project administration, Funding acquisition, Conceptualization. **Jussi Rossi:** Writing – original draft, Visualization, Software, Investigation, Formal analysis, Data curation.

## Declaration of Competing Interest

The authors declare the following financial interests/personal relationships which may be considered as potential competing interests: Jussi Rossi reports financial support was provided by Research Council of Finland. Jussi Rossi reports financial support was provided by Finnish Cultural Foundation. If there are other authors, they declare that they have no known competing financial interests or personal relationships that could have appeared to influence the work reported in this paper.

## Data Availability

Data will be made available on request. Data underlying the results presented in this paper are not publicly available at the time but may be obtained from the authors upon reasonable request.
